# GLIM criteria for the diagnosis of malnutrition in patients with cirrhosis: A narrative review

**DOI:** 10.1002/jpen.70095

**Published:** 2026-04-29

**Authors:** Bárbara Chaves Santos, Ana Teresa Limon‐Miro, Puneeta Tandon, Maria Isabel Toulson Davisson Correia, Carla M. Prado, Maria Cristina Gonzalez, Lucilene Rezende Anastácio

**Affiliations:** ^1^ Graduate Program in Food Science Universidade Federal de Minas Gerais Belo Horizonte Brazil; ^2^ Department of Agricultural, Food & Nutritional Science University of Alberta Edmonton Alberta Canada; ^3^ Department of Medicine, Division of Gastroenterology (Liver Unit) University of Alberta Edmonton Alberta Canada; ^4^ Surgery Graduate Program Universidade Federal de Minas Gerais Belo Horizonte Brazil; ^5^ Graduate Program in Nutrition and Food Universidade Federal de Pelotas Pelotas Brazil

**Keywords:** GLIM, liver cirrhosis, malnutrition, nutritional assessment, validation

## Abstract

**Background:**

This narrative review aims to describe and discuss the validity of the Global Leadership Initiative on Malnutrition (GLIM) criteria in patients with cirrhosis.

**Methods:**

The literature search was performed in PubMed and EMBASE between May and August 2024 and updated in January 2025, including original studies published in English in which patients with cirrhosis were assessed according to the GLIM framework.

**Results:**

Seventeen studies with data from 4275 patients were included. There were substantial differences in the use of the GLIM criteria among studies. Overall, the prevalence of malnutrition by the GLIM framework when all the criteria were considered ranged between 21% and 65.1% and was frequently lower when compared to the prevalence by other tools. Sensitivity and specificity of the GLIM criteria in comparison to standard tools were tested in four studies, and none achieved the minimum requirements to determine adequate concurrent validity. In contrast, the diagnosis of malnutrition by the framework demonstrated adequate predictive validity in six studies (HR/OR 2.1–7.2).

**Conclusion:**

In patients with cirrhosis, the prevalence of malnutrition is generally lower when assessed using the GLIM criteria compared with standard tools. Evidence on concurrent validity is currently limited, with existing studies showing insufficient sensitivity and specificity in relation to the Subjective Global Assessment. However, malnutrition defined by GLIM independently predicts adverse clinical outcomes in this population. Future research should focus on the current gaps and on the adequate use of the criteria to strengthen their clinical applicability in nutritional assessment and management of cirrhosis.

## INTRODUCTION

Different tools have been used for nutritional assessment of patients with cirrhosis.^1^ This includes widely used instruments, such as the Subjective Global Assessment (SGA)^2^ and others developed specifically for this population, such as the Royal Free Hospital Global Assessment (RFH‐GA).^3^ Despite the availability of validated and even specific tools, nutritional assessment is still challenging in these patients, mainly in those with advanced disease and ascites, which hampers the assessment of their current body weight and muscle mass. Also, the prevalence of malnutrition varies according to the tool used for diagnosis,^4^ making it difficult to establish the actual prevalence in this population, which is currently estimated to range from 20%–60%.^5^

Due to the lack of consensus regarding the most appropriate nutritional assessment tool for use in clinical settings, the Global Leadership Initiative on Malnutrition (GLIM) framework was designed to standardize the diagnosis of malnutrition worldwide.^6^, ^7^ Following the initial publication of these criteria,^6^, ^7^ many researchers tested their validity in different clinical settings and specific populations.^8^ Considering the particular characteristics of patients with cirrhosis, the applicability of the GLIM criteria in this population should be carefully evaluated, as results may differ from those with other clinical conditions.

The GLIM framework is a two‐step process.^6^, ^7^ The first involves screening for nutritional risk using a validated tool. After that, patients at nutritional risk are assessed for malnutrition, considering three phenotypic and two etiologic criteria. Phenotypic criteria include unintentional weight loss, low body mass index (BMI), and low muscle mass. Etiologic criteria comprise reduced food intake and disease burden/inflammation. All five criteria should be assessed, and patients presenting at least one phenotypic and one etiologic criterion are classified as malnourished. The severity of malnutrition can be further defined by assessing the phenotypic criteria. The initial consensus report was published in 2019,^6^, ^7^ and was followed by a guidance on validation of the criteria,^9^, ^10^ on the assessment of muscle mass,^11^, ^12^ and on inflammation^13^, ^14^ criteria, and a 5‐year update.^15^, ^16^

The GLIM criteria can be validated by assessing their concurrent or predictive validity.^9^, ^10^ Concurrent validity consists of performing a diagnosis of malnutrition using a gold or semi‐gold standard tool while the GLIM criteria are applied. Next, the sensitivity and specificity values can be calculated and are considered satisfactory when both values are >80%.^9^, ^10^ Predictive validity is an analysis of the prognostic impact of malnutrition diagnosis based on the GLIM criteria on relevant clinical outcomes. As malnutrition is widely recognized as a risk factor for negative clinical outcomes,^6^, ^7^ this association should be identified when a diagnosis is performed using these criteria. According to the guidance on validation of the GLIM criteria,^9^, ^10^ for predictive validation a Hazard Ratio (HR) or Odds Ratio (OR) ≥ 2.0 is considered satisfactory.^9^, ^10^

As the GLIM framework was proposed to standardize the diagnosis of malnutrition, the criteria must be tested in different populations to assess their performance. Since there is no consensus on the most appropriate tool for diagnosing malnutrition in patients with cirrhosis, there is a clinical need to assess the validity of the GLIM criteria in this population, especially considering the challenges in assessing the nutritional status in this population. The main purpose of this review was to gather data available in the literature regarding the use of the GLIM criteria in patients with cirrhosis and critically assess the applicability and validity of the framework in this population.

## MATERIALS AND METHODS

This narrative review was written following the criteria specified in the Scale for the Assessment of Narrative Review Articles (SANRA).^17^ The literature search was performed between May and August 2024, and updated between December 2024 and January 2025, in the databases PubMed and EMBASE, using the following search terms: (“GLIM” OR “Global Leadership Initiative on Malnutrition”) AND (“cirrhosis” OR “liver transplant” OR “liver disease”). Inclusion criteria were cross‐sectional, prospective, and retrospective studies written in English, with a sample including patients with cirrhosis, and nutritional assessment according to the GLIM criteria, regardless of the phenotypic and etiologic criteria used or the publication date. Duplicates, studies with largely overlapping cohorts, and review papers were excluded. After the screening of titles and abstracts, the full‐text assessment of the studies was performed. A single reviewer (BCS) was responsible for all steps. In case of overlapping cohorts, studies were selected based on the most complete and/or well‐described application of the criteria or based on the largest sample size.

The following data were extracted from studies that met the inclusion and exclusion criteria: authors, country, study design, sample size, age and sex distribution of the sample, inclusion and exclusion criteria, primary and secondary aims, nutritional screening tool if applicable, standard nutritional assessment tool if applicable, GLIM phenotypic and etiologic criteria, prevalence of malnutrition according to GLIM and standard tools, results regarding concurrent and predictive validity, and additional relevant information.

As the GLIM criteria continue to evolve,^15^, ^16^ with updates to validation requirements^9^, ^10^ and accepted parameters^11^, ^12^, ^13^, ^14^ since their initial publication in 2019,^6^, ^7^ we opted to include all studies reporting using the GLIM criteria for malnutrition diagnosis, regardless of the specific criteria applied. At the time of the original publication,^6^, ^7^ handgrip strength was considered a valid supportive measure within the phenotypic criterion of low muscle mass. Therefore, we did not exclude studies in which handgrip strength was used as a surrogate measure for classifying low muscle mass, especially considering the limited availability of accurate assessment methods for patients with cirrhosis.^18^, ^19^, ^20^ However, it is important to note that this approach is no longer accepted, as clarified in the 2022 guidance.^11^, ^12^ Also, while prospective studies are now expected to assess all phenotypic and etiologic criteria, retrospective validation studies were allowed to use at least one phenotypic and one etiologic criterion.^9^, ^10^ Authors were also encouraged to test different combinations of individual criteria.^9^, ^10^ Current guidance emphasizes that all criteria must be evaluated to rule out a diagnosis of malnutrition.^15^, ^16^ Considering this context, we included all studies applying the GLIM criteria for malnutrition diagnosis in patients with cirrhosis to provide a broad and inclusive overview of how the tool has been operationalized in this population. Our summary of results was stratified according to whether the criteria were applied as per current recommendations.

## RESULTS

**Figure 1:**
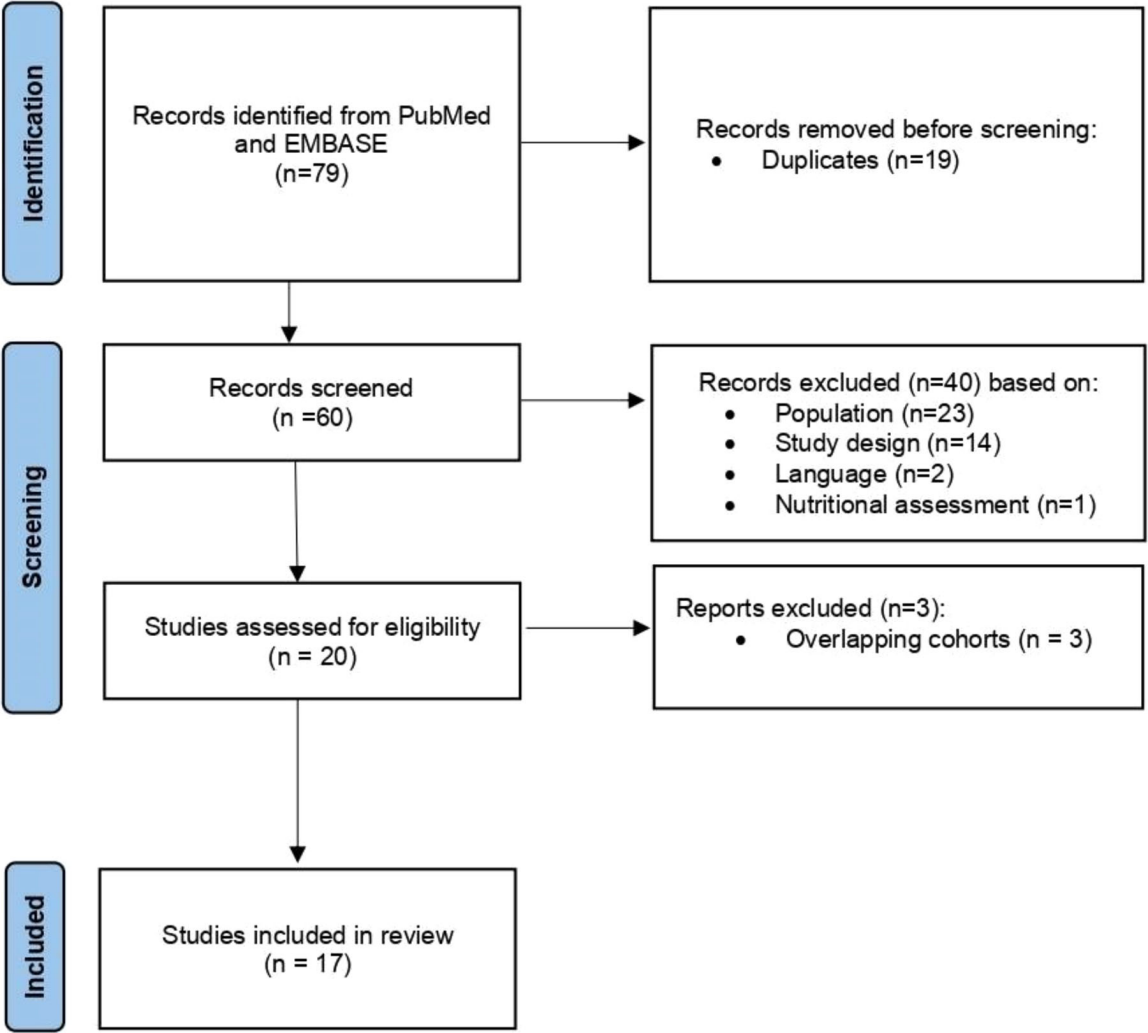
Flow chart of the search and inclusion of the studies.

The search retrieved 79 results (Figure 1). After applying the inclusion and exclusion criteria, seventeen studies with a total sample of 4275 patients were included in this review.

The general description of the studies included is presented in Table 1. Most studies were carried out in China (*n* = 8),^18^, ^21^, ^22^, ^23^, ^24^, ^25^, ^26^, ^27^ followed by Brazil (*n* = 4),^19^, ^20^, ^28^, ^29^ and the individual sample sizes varied from 63–1002. The mean age of the patients was 59 years, ranging from 52–74 years. Regarding the clinical setting, the majority of the studies (*n* = 9) included only hospitalized patients.^18^, ^21^, ^22^, ^23^, ^24^, ^25^, ^26^, ^27^, ^30^ When considering disease severity, authors of five studies included only patients with decompensated disease or those on the waiting list for a liver transplant,^18^, ^19^, ^25^, ^29^, ^31^ and in the remaining studies, consecutive patients with cirrhosis were included regardless of disease severity. In one study, patients with chronic liver disease (42% without cirrhosis) were included.^32^

**Table 1:** Characteristics of included studies in the narrative review evaluating GLIM criteria to diagnose malnutrition in patients with cirrhosis.

First author, year	Country	Study design	Sample size	Age (years)^a^ and sex distribution (% males)	Clinical setting and severity	Inclusion criteria	Exclusion criteria
Bannert et al. (2023)^30^	Germany	Multicenter cross‐sectional	64	57.5 ± 10.4 67%	Inpatient	Diagnosis of cirrhosis; Age ≥18 years	Parenteral nutrition in the past 6 months; Ongoing nutritional intervention for >7 days; TIPS; Previous LTx; Pregnancy/lactation; Pacemaker/implanted defibrillator; Malignant disease in the past 3 years
Boulhosa et al. (2020)^28^	Brazil	Cross‐sectional	166	56.7 ± 13.4 68.1%	Outpatient and inpatient, MELD‐Na 14 (11;17)	Advanced chronic liver disease of different etiologies; Age >18 years; Evaluated within the first 48 h post‐admission or at the first nutritional appointment	Cancer diagnosis or AIDS; Using orthopedic prosthesis; Unable to perform the nutritional assessment
Casas Deza et al. (2021)^34^	Spain	Prospective	63	63.1 ± 9.9 61.9%	Outpatient MELD 10.5 ± 3.6	All consecutive patients with a diagnosis of cirrhosis	Cancer diagnosis; Heart failure; Pulmonary hypertension; Active infection; Enteral nutrition; Age <18 years; Severe mental or psychiatric condition that could interfere with the study
Fonseca et al. (2023)^29^	Brazil	Retrospective	419	52 (46; 59) 69.2%	Outpatient MELD‐Na 17 (13.7; 20.0)	Advanced cirrhosis on the waiting list for LTx; Age >18 years	Indication for multiorgan transplant or retransplant; Pregnancy/lactation
Guo et al. (2023)^18^	China	Prospective	184	64 (57; 69) 45.1%	Inpatient MELD‐Na 9 (7; 12)	Aged ≥18 years; Confirmed diagnosis of cirrhosis; Admission due to cirrhosis‐associated complications; Abdominal CT images within 3 months prior to hospitalization/during hospitalization and concomitant evaluation of HGS within 48 h after admission	Acute‐on‐chronic liver failure on admission; Severe hepatic encephalopathy; Primary HCC or extra‐hepatic malignancies; LTx; Refusal to regular follow‐up
He et al. (2024)^21^	China	Prospective	125	57.9 ± 13.6 64.8%	Inpatient	Diagnosis of cirrhosis; Aged >18 years; CT scan of the abdomen or chest (L3 or T12 images)	Tuberculosis; AIDS; Malignancies other than HCC; Pregnancy/lactation; Acute‐on‐chronic liver failure on admission; Severe cognitive problems; LTx
Jiang et al. (2024)^22^	China	Retrospective	1,002	56.3 ± 12.8 63.1%	Inpatient MELD 17.7 ± 7.7	Adults with cirrhosis; Abdominal CT examination during hospitalization	Confirmed or strongly suspected diagnosis of malignant tumors; Long‐term bedridden; AIDS; Pregnancy/lactation; Chronic renal and/or respiratory insufficiency; Severe heart disease; Any condition that impair nutrient absorption
Li et al. (2024)^23^	China	Retrospective	360	60.5 ± 9.8 75.0%	Inpatient MELD 14.5 ± 5.7	Adults with a clinical diagnosis of cirrhosis and HCC; Abdominal CT examination during hospitalization	Incomplete clinical information; Confirmed or strongly suspected diagnosis of malignant tumors other than HCC; Long‐term bedridden; AIDS; Pregnancy/lactation; Chronic renal and/or respiratory insufficiency; Severe heart disease; Conditions that cause intestinal nutrient absorption disorders
Lindqvist et al. (2019)^31^	Sweden	Retrospective	133	59 (19) 73%	Outpatient MELD 12 (7)	Adults with chronic liver disease under evaluation for LTx	Age <18 years; Language difficulties; Severe hepatic encephalopathy
Miwa et al. (2022)^32^	Japan	Retrospective	406	74 (66; 79) 67%	Outpatient MELD 8 (7; 10)	Aged ≥20 years; Diagnosed with chronic liver disease of any etiology; Assessed for malnutrition	Non‐hepatic active malignancies; History of organ transplantation; LTx during the follow‐up period; Life‐threatening comorbidities
Petric et al. (2023)^35^	Slovenia	Retrospective	138	57.5 (range: 22–69) 72.5%	N/A MELD 14 ± 7	Adults who had the first orthotropic LTx from brain‐dead donors	Re‐transplantation; Abdominal CT scans could not be obtained or received
Santos et al. (2021)^41^	Brazil	Retrospective	152	52 (46.5; 59.5) 66.4%	Outpatient MELD‐Na 17 (14.2; 20.3)	Patients with cirrhosis who were on the waiting list for LTx; Aged >20 years; Regular follow‐up at the outpatient clinic	Indication for multi‐organ transplant or re‐transplant; Pregnancy/lactation; Hypothyroidism or hyperthyroidism
Sousa et al. (2023)^20^	Brazil	Prospective	126	52.8 ± 12.3 56.3%	Outpatient MELD‐Na 16.1 ± 5.4	Patients with chronic liver disease who met the Milan criteria; Aged >18 years; Both sexes; Awaiting LTx	Unable to undergo any nutritional assessment; Sensory impairment; Pregnancy; Loss of follow‐up in the outpatient clinic; Death due to reasons unrelated to liver disease
Wu et al. (2022)^24^	China	Prospective	109	56.4 ± 10.4 71.6%	Inpatient MELD 9.5 (8; 13)	Hospitalized patients; Age >18 years; Diagnosis of cirrhosis	Unstable renal function; Diabetes; Malignant tumors; Any other diseases that could affect metabolism; Refusal to participate
Yang et al. (2023)^25^	China	Prospective	387	64 (range: 57–69) 48.6%	Inpatient MELD‐Na 9.5 (6.9; 12.5)	Consecutively hospitalized patients; Aged ≥18 years; With cirrhosis and signs of decompensation	Acute‐on‐chronic liver failure; Hepatic/extrahepatic cancers; LTx, No regular follow‐up visits; Severe cognitive problems
Yang et al. (2023)^26^	China	Cross‐sectional	363	64 (57; 69) 48.8%	Inpatient MELD‐Na 9.5 (6.9; 12.5)	Adult patients with cirrhosis who were hospitalized	Acute‐on‐chronic liver failure upon admission; Intrahepatic or extrahepatic malignancies; Refusal to follow‐up; LTx; Severe cognitive problems
Zhang et al. (2023)^27^	China	Cross‐sectional	78	54.5 ± 12.5 64.1%	Inpatient MELD 10.1 ± 5.1	Patients with cirrhosis who were admitted to the hospital; Abdominal CT examination	Severe cardiopulmonary disease; Cancer; Inflammatory bowel disease; Any other metabolic disorders; Age over 80 and under 18 years

Abbreviations: AIDS, Acquired Immunodeficiency Syndrome; CT, computed tomography; HCC, hepatocellular carcinoma; HGS, handgrip strength; IQR, interquartile range; LTx, liver transplantation; L3, third lumbar vertebra; MELD, model for end‐stage liver disease; MELD‐Na, model for end‐stage liver disease–sodium; N/A, not available; TIPS, transjugular intrahepatic portosystemic shunt; T12, twelfth thoracic vertebra.

^a^ Age is presented as mean ± sd or median (IQR).

A description of how the GLIM framework was applied, and the prevalence of malnutrition in each study is reported in Table 2. A nutritional screening tool was applied as the first step of the GLIM framework in six studies,^18^, ^20^, ^22^, ^23^, ^25^, ^26^ and in four of these studies^18^, ^20^, ^25^, ^26^ the Royal Free Hospital Nutrition Prioritizing Tool (RFH‐NPT) was used.^33^ For the standard nutritional assessment tool, the SGA was used in six studies,^19^, ^22^, ^23^, ^24^, ^29^, ^31^ and both the SGA and the RFH‐GA were used in two studies.^20^, ^32^ Regarding the application of the GLIM criteria, all the phenotypic criteria were assessed in fifteen studies.^18^, ^19^, ^20^, ^22^, ^23^, ^24^, ^25^, ^26^, ^27^, ^28^, ^29^, ^30^, ^31^, ^32^, ^34^ The etiologic criterion of disease burden/inflammation was assessed in all studies, and the food intake was assessed in twelve studies.^19^, ^20^, ^21^, ^22^, ^23^, ^25^, ^26^, ^28^, ^29^, ^30^, ^32^, ^34^ Authors of eleven studies assessed all the phenotypic and etiologic criteria.^19^, ^20^, ^22^, ^23^, ^25^, ^26^, ^28^, ^29^, ^30^, ^32^, ^34^ Malnutrition severity was classified in only two studies.^31^, ^34^

**Table 2:** Application of the GLIM criteria in patients with cirrhosis and main results of studies included in the narrative review.

First author, year	Nutritional screening	Standard nutritional assessment tool	GLIM criteria						Prevalence of malnutrition—GLIM	Prevalence of malnutrition—standard tool
			WL	Low BMI	Low MM	Inflammation	Cirrhosis	FI		
**Diagnosis was performed as recommended by the GLIM consensus**										
Bannert et al. (2023)^30^	NRS‐2002, MUST, and RFH‐NPT^a^	N/A	X	X	X (BIA)	X (CRP)	X	X	GLIM: 61% GLIM‐CRP: 59%	N/A
Boulhosa et al. (2020)^28^	NRS‐2002 and RFH‐NPT^a^	N/A	X	X	X (MAMA)	X (CRP)	–	X	57.2%	N/A
Casas Deza et al. (2021)^34^	RFH‐NPT, MNA‐SF, and LDUST^a^	N/A	X	X	X (BIA)	X (CRP or decompensation)	–	X	Total: 38.1% Moderate: 15.9% Severe: 22.2%	N/A
Fonseca et al. (2023)^29^	N/A	SGA	X	X	X (MAMC)	X (MELD, MELD‐Na, Child‐Pugh score, serum albumin)	X	X	54.4%. Different combinations ranged between 3.1% and 58.2%	68.8%
Guo et al. (2023)^18^	RFH‐NPT	N/A	X	X	X (CT or HGS)	–	X	–	GLIM‐CT: 34.2% GLIM‐HGS: 42.4%	N/A
Jiang et al. (2024)^22^	NRS‐2002	SGA	X	X	X (CT)	X (serum albumin)	–	X	34.3%	86%
Li et al. (2024)^23^	NRS‐2002	SGA	X	X	X (CT)	X (serum procalcitonin)	–	X	49.7%	80.3%
Lindqvist et al. (2019)^31^	N/A	SGA	X	X	X (DXA or MAMC)	–	X	–	Total: 32% Severe: 12%	Moderate: 23% Severe: 8%
Miwa et al. (2022)^32^	N/A	SGA and RFH‐GA	X	X	X (BIA)	X (CRP)	–	X	21%	SGA: 35% RFH‐GA: 26%
Sousa et al. (2023)^20^	RFH‐NPT	SGA and RFH‐GA	X	X	X (two of the following three criteria: MAMC, MAC, and HGS)	X (both of the following criteria: Child‐Pugh score and MELD‐Na)	–	X	56.3%	SGA: 62.7% RFH‐GA: 85.7%
Wu et al. (2022)^24^	N/A	SGA	X	X	X (MAMC)	–	X	–	65.1%	55.0%
Yang et al. (2023)^25^	RFH‐NPT	N/A	X	X	X (CT)	X (NLR)	–	X	28.7%	N/A
Yang et al. (2023)^26^	RFH‐NPT	N/A	X	X	X (CT)	X (NLR)	–	X	36.4%. Different combinations: ranged between 13.6% and 42.4%	N/A
Zhang et al. (2023)^27^	NRS‐2002, RFH‐NPT, and LDUST^a^	N/A	X	X	X (CC)	–	X	–	42.3%	N/A
**Missing phenotypic or etiologic criteria or diagnosis was not performed as recommended by the GLIM consensus**										
He et al. (2024)^21^	NRS‐2002 and RFH‐NPT^a^	N/A	X	X	–	X (CRP)	–	–	59.2%	N/A
Petric et al. (2023)^35^	N/A	N/A	–	–	X (CT)	–	X	–	N/A	N/A
Santos et al. (2021)^41^	N/A	SGA	X	X	X (BIA‐FFMI or MAMA or HGS or BIA‐PhA)	X (MELD or MELD‐Na or Child‐Pugh score or hypermetabolism or serum albumin)	–	X	Ranged between 0.7% and 30.9%	63.2%

Abbreviations: BIA, bioelectrical impedance analysis; BMI, body mass index; CRP, C‐reactive protein; CT, computed tomography; DXA, dual energy X‐ray absorptiometry; FI, food intake; GLIM, global leadership initiative on malnutrition; LDUST, liver disease undernutrition screening tool; MAC, mid‐arm circumference; MAMC, mid‐arm muscle circumference; MELD, model for end‐stage liver disease; MELD‐Na, model for end‐stage liver disease‐sodium; MM, muscle mass; MUST, malnutrition universal screening tool; NLR, neutrophil‐to‐lymphocyte ratio; NRS‐2002, nutritional risk screening 2002; N/A, not available; PhA, phase angle; RFH‐GA, Royal Free Hospital–Global Assessment; RFH‐NPT, Royal Free Hospital‐Nutritional Prioritizing Tool; SGA, subjective global assessment; WL, weight loss

^a^ It is unclear whether the nutritional screening was applied as the first step of the GLIM framework.

Regarding the phenotypic criteria, authors of twelve studies calculated dry weight to classify weight loss and low BMI. For the assessment of the low muscle mass phenotypic criterion, anthropometry was used in the majority of the studies (*n* = 7): mid‐arm muscle circumference,^20^, ^24^, ^28^, ^29^, ^31^ mid‐arm muscle area,^19^ mid‐arm circumference^20^ or calf circumference,^27^ followed by bioelectrical impedance analysis (BIA) in four studies,^19^, ^30^, ^32^, ^34^ six using computed tomography,^18^, ^22^, ^23^, ^25^, ^26^, ^35^ and one using dual‐energy X‐ray absorptiometry.^31^ Authors of four studies included the assessment of handgrip strength within the GLIM criteria.^18^, ^19^, ^20^, ^34^ The muscle mass phenotypic criterion was not assessed in one study.^21^ In regard to the etiologic criterion of disease burden/inflammation, diagnosis of cirrhosis was considered in most studies,^18^, ^24^, ^27^, ^29^, ^30^, ^35^ followed by model for end‐stage liver disease (MELD), MELD‐sodium or Child‐Pugh values,^19^, ^20^, ^29^ C‐reactive protein values,^21^, ^30^, ^32^ serum albumin values,^19^, ^22^, ^29^ neutrophil‐to‐lymphocyte ratio,^25^, ^26^ serum procalcitonin values,^23^ or hypermetabolism.^19^

The prevalence of malnutrition according to the GLIM criteria, considering all studies, ranged between 0.7%–65.1%, with a lower prevalence in studies testing different combinations of individual phenotypic and etiologic criteria, especially those with low BMI as the phenotypic criterion.^19^, ^21^, ^29^, ^35^ When only the studies assessing all the phenotypic and etiologic criteria were considered, the prevalence ranged between 21.0%–65.1%.^18^, ^20^, ^22^, ^23^, ^24^, ^25^, ^26^, ^27^, ^28^, ^29^, ^30^, ^31^, ^32^, ^34^ The prevalence, as reported by the SGA, ranged between 31%–86%, and by the RFH‐GA, between 26%–85.7%. Authors of two of the included studies assessed the nutritional status considering both SGA and RFH‐GA as the standard,^20^, ^32^ with opposite results. In the study by Miwa and collaborators, the prevalence was higher by SGA,^32^ while Sousa and collaborators found a higher prevalence for the RFH‐GA.^20^ Overall, the prevalence of malnutrition was higher when using the SGA or RFH‐GA tools compared to the GLIM criteria, except for one study.^24^

Authors of five studies compared the diagnosis of malnutrition by the GLIM criteria using the SGA as the semi‐gold standard,^22^, ^23^, ^24^, ^29^, ^32^ and four of them reported parameters to assess the sensitivity and specificity values (Table 3), although confidence intervals were not provided. Fonseca and collaborators^29^ tested different combinations of the phenotypic and etiologic criteria, as well as the diagnosis considering any phenotypic plus any etiologic criteria and any phenotypic plus the diagnosis of cirrhosis as the etiologic criterion. Although the sensitivity and specificity values did not meet the recommended thresholds (>80%), the overall values were above 65% for both combinations. When using the diagnosis of cirrhosis as the etiologic criterion (i.e., all patients had the etiologic criterion and were classified as malnourished when any of the phenotypic criteria were met), there was an increase in sensitivity and a reduction in specificity when compared to the combination in which food intake and disease severity were assessed. All the other studies^23^, ^24^, ^32^ reporting sensitivity and specificity considered any combination of at least one phenotypic and one etiologic criterion, and none of them identified adequate concurrent validity.

**Table 3:** Concurrent validity of the GLIM criteria in patients with cirrhosis (*n* = 5 studies).

Study	Standard tool	Sensitivity	Specificity	Kappa
Fonseca et al.^29^	SGA			
*PHEN_cirrhosis* a		69.2	65.4	N/A
*PHEN_ETIO* b		66.8	72.3	N/A
Jiang et al.^22^	SGA	N/A	N/A	0.118
Li et al.^23^,^c^	SGA	57.4	81.6	0.069
Miwa et al.^32^,^d^	SGA	65.1	92.9	N/A
Wu et al.^24^,^e^	SGA	89.5	73.1	0.539

Abbreviations: N/A, not available; SGA, subjective global assessment.

^a^ Any phenotypic criteria + diagnosis of cirrhosis (all patients).

^b^ Any phenotypic criteria + any etiologic criteria.

^c^ Sensitivity and specificity calculated from data in table 3.

^d^ Sensitivity and specificity calculated from data in table 1.

^e^ Sensitivity and specificity calculated from data in table 3.

**Figure 2:**
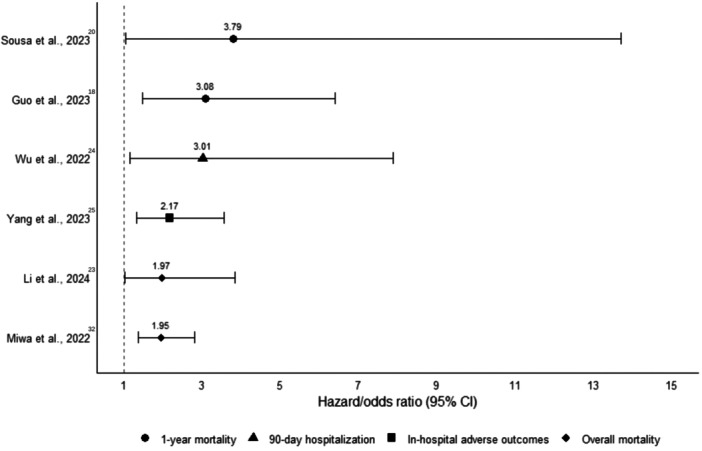
Multivariate HR/OR from studies assessing the impact of malnutrition according to the GLIM criteria on clinical outcomes in patients with cirrhosis.

Regarding the predictive validity (Table 4), the impact of malnutrition assessed by the GLIM criteria on the incidence of mortality was evaluated in six studies,^18^, ^19^, ^20^, ^23^, ^29^, ^32^ and authors of three studies evaluated the predictive effect on length of hospital stay,^19^ re‐hospitalization,^24^ and other clinical outcomes.^25^ For the studies in which the HR or OR values were reported, malnutrition according to the GLIM criteria was associated with an increased risk (HR ≥ 2) of mortality in four studies,^18^, ^19^, ^20^, ^29^ longer hospital length of hospital stay (OR ≥ 2) after liver transplant in one study,^19^ higher risk (HR ≥ 2) of 90‐day re‐hospitalization in one study,^24^ and higher risk (HR ≥ 2) of in‐hospital adverse outcomes in one study.^25^ The HR and OR values of the studies in which all the phenotypic and etiologic criteria were applied are demonstrated in Figure 2.

**Table 4:** Predictive validity of the GLIM criteria and different combinations of individual phenotypic and etiologic criteria in patients with cirrhosis.

Study	Outcome	Adjusted OR/HR	95% CI
Fonseca et al.^29^	1‐year mortality		
*MAMC/MELDNa*		2.42	1.42; 4.12
*MAMC/MELD*		2.38	1.43; 3.96
*BMI or MAMC/MELDNa*		2.38	1.40; 4.04
*BMI or MAMC/MELD*		2.35	1.41; 3.89
*WL/ALB*		2.23	1.14; 4.34
*WL or MAMC/MELDNa*		2.21	1.31; 3.74
*PHEN/MELDNa*		2.14	1.27; 3.62
*WL or MAMC/MELD*		2.12	1.28; 3.52
*PHEN/MELD*		2.10	1.24; 3.43
Guo et al.^18^	1‐year mortality		
*GLIM/CT*		3.08	1.48; 6.39
*GLIM/HGS*		2.42	1.18; 5.00
Li et al.^23^	Overall mortality (up to 5‐year follow‐up)	1.97	1.01; 3.84
Miwa et al.^32^	Mortality (0.8–3.5 years)	1.95	1.37; 2.81
Santos et al.^41^	Mortality before and up to 1‐year after LTx		
*PhA/MELD*		2.08	1.09; 3.97
*PhA/MELDNa*		2.17	1.14; 4.13
*PhA/Child‐Pugh*		1.96	1.02; 3.77
	Hospital LOS after LTx		
*HGS/Child‐Pugh*		7.21	1.22; 42.50
Sousa et al.^20^	1‐year mortality	3.79	1.05; 13.70
Wu et al.^24^	90‐days re‐hospitalization	3.01	1.14; 7.87
Yang et al.^25^	In‐hospital adverse outcomes	2.17	1.32; 3.56

Abbreviations: ALB, serum albumin; BMI, body mass index; CI, confidence interval; CT, computed tomography; GLIM, Global Leadership Initiative on Malnutrition; HGS, handgrip strength; HR, hazard ratio; LOS, length of stay; LTx, liver transplant; MAMC, mid‐arm muscle circumference; MELD‐Na, model for end‐stage liver disease Sodium; OR, odds ratio; PhA, phase angle; PHEN, phenotypic criteria; WL, weight loss.

## DISCUSSION

Since the publication of the GLIM consensus report in 2019, a few studies have reported its application in patients with cirrhosis.^18^, ^19^, ^20^, ^21^, ^22^, ^23^, ^24^, ^25^, ^26^, ^27^, ^28^, ^29^, ^30^, ^31^, ^32^, ^34^, ^35^ As the criteria's validity gains support in various contexts and potentially enters clinical practice, it is relevant to discuss its applicability in specific groups. Overall, authors of a limited number of studies (*n* = 4) have assessed concurrent validity as recommended by the GLIM working group^9^, ^10^ in this population, and none of them demonstrated satisfactory validity considering the SGA as the standard.^23^, ^24^, ^29^, ^32^ Regarding predictive validity, mortality was the most common clinical outcome,^18^, ^19^, ^20^, ^23^, ^29^, ^32^ and malnutrition has demonstrated strong predictive validity in this population.

When assessing the GLIM phenotypic criteria, it is important to emphasize that fluid overload (ascites and/or edema) is one of the most common complications of advanced liver disease.^1^ Dry body weight can be estimated by subtracting a percentage of weight according to the severity of the ascites (mild 5%, moderate 10%, severe 15%) or edema (5% if bilateral lower limb edema), and dry‐BMI can be further calculated. However, this approach is still not validated.^36^ In patients with ascites, nutritional assessment is hampered due to the uncertainty in assessing the current weight and weight loss. Also, accuracy is reduced for the most commonly used tools to assess the muscle mass.^36^ Therefore, the three GLIM phenotypic criteria may have limitations for patients with decompensated liver disease. In all of the studies reporting the prevalence for each phenotypic criterion, low BMI was the least prevalent,^19^, ^20^, ^24^, ^28^, ^29^, ^30^ even though dry‐BMI was used in most studies. It is widely known that the BMI is not an adequate parameter to identify malnutrition in clinical settings, since it does not distinguish between the different components of body weight.^37^ A scoping review on the utilization of the GLIM criteria demonstrated that low BMI was the most common phenotypic criterion so far, and was used as the only phenotypic criterion in a high percentage of studies.^8^ The authors of the scoping review suggested that future validation studies should analyze this criterion in light of its limitations.^8^ It is also important to emphasize that, even though BMI and weight loss may be easier to assess in most settings when compared to muscle mass, all three phenotypic criteria should be assessed when applying the GLIM framework.^15^, ^16^

Mandatory objective assessment of muscle mass is an important feature of the GLIM framework, since the loss of muscle is one of the main components of malnutrition and has a strong negative prognostic impact.^9^, ^10^ In patients with cirrhosis, accurate assessment of muscle mass within the GLIM criteria is even more important due to limitations in assessing the other two phenotypic criteria, as discussed above. In six of the included studies, reduced muscle mass was the most prevalent phenotypic criterion (compared to weight loss and low BMI).^19^, ^23^, ^24^, ^28^, ^29^, ^32^ Nevertheless, researchers and health professionals should be aware that some methods used to estimate muscle mass or other compartments (e.g., fat‐free mass index, appendicular lean soft tissue index) may have limited accuracy in patients with fluid retention, as this clinical population.^1^ Anthropometry was used to assess the muscle mass in the majority of the studies,^19^, ^20^, ^24^, ^27^, ^28^, ^29^, ^31^ as these are simple and inexpensive assessments, frequently requiring only a measuring tape and caliper. Anthropometry is considered one of the clinical approaches to estimate muscle mass according to the GLIM Working Group, and is acceptable as part of the criteria when the technical approaches are not available.^11^, ^12^ Within the anthropometric measurements, mid‐arm muscle circumference seems to be an adequate parameter for the assessment of patients with cirrhosis since it is not affected when patients have ascites or lower limb edema.^36^ Fonseca and collaborators observed that reduced mid‐arm muscle circumference and the presence of ascites were the criteria most associated with malnutrition by SGA in a machine‐learning model.^29^ Therefore, mid‐arm muscle circumference seems to be the best bedside option to estimate muscle mass in settings where technology‐based methods are not available.^11^, ^12^

Besides anthropometry, different tools were used to assess muscle mass within the GLIM framework in patients with cirrhosis. Authors of four studies used BIA to estimate the fat‐free mass index^19^, ^30^, ^34^ and appendicular skeletal muscle index^32^ compartments. However, BIA has limited accuracy in patients with fluid retention, since the parameters are estimated by equations and can be overestimated in these patients.^11^, ^12^, ^38^, ^39^ Some of the included studies used dual‐energy X‐ray absorptiometry^31^ or computed tomography,^18^, ^22^, ^25^, ^26^, ^35^ considered as technical approaches, to assess muscle mass within GLIM. These are accurate methods to assess the lean soft tissue and muscle mass, respectively, and should therefore be given priority for body composition assessment according to the GLIM Working Group.^11^, ^12^ Still, authors should be aware that the results may also be influenced by fluid retention,^39^, ^40^, ^41^ and validated cutoff points should be used to classify low muscle mass.^11^, ^12^

When analyzing the etiologic criteria, authors used different parameters to classify disease burden/inflammation, and these discrepancies may have affected the malnutrition diagnosis and validation of the GLIM criteria. For the disease burden/inflammation criterion, some acute and chronic diseases that are known to lead to some degree of inflammation can be sufficient to fulfill the etiologic criterion within the GLIM framework.^13^, ^14^ Therefore, some authors assessed only the phenotypic criteria to diagnose malnutrition.^18^, ^24^, ^27^, ^29^, ^30^, ^35^ Considering all patients with cirrhosis as having the inflammation etiologic criterion for malnutrition may increase the sensitivity of the framework, but specificity could be negatively affected, as demonstrated by Fonseca and collaborators.^29^ The GLIM working group recognizes that cirrhosis is one of the conditions usually associated with mild to moderate chronic inflammation, and when laboratory tests are available, C‐reactive protein levels should be measured to confirm the presence of inflammation in uncertain cases. However, these values may be lower in patients with advanced liver disease.^13^, ^14^ Furthermore, food intake was not assessed in some studies in which all patients with cirrhosis were automatically classified as meeting the inflammation etiologic criterion.^18^, ^24^, ^27^, ^31^ Although the diagnosis of cirrhosis may meet this criterion from an operational standpoint, assessing food intake remains essential to accurately identify the factors contributing to malnutrition. Given that reduced food intake is common in patients with cirrhosis,^42^, ^43^, ^44^ evaluating this parameter is crucial to ensure an accurate nutritional diagnosis and to guide appropriate nutritional care.

Authors of six out of the seventeen studies applied a nutritional screening tool and proceeded with nutritional assessment in those patients who were at nutritional risk.^18^, ^20^, ^22^, ^23^, ^25^, ^26^ The majority of these studies (*n* = 5) involved hospitalized patients. Although we recognize the importance of nutritional screening as part of the GLIM framework, we did not consider the lack of this step as a limitation or exclusion criterion for this review. Particularly in outpatient settings and when evaluating patients diagnosed with conditions known to increase nutritional risk (e.g., cirrhosis), nutritional screening may be bypassed in clinical practice, since time and resources are prioritized to perform in‐depth nutritional assessments. Future studies should evaluate whether nutritional screening is imperative when applying the GLIM criteria in patients with cirrhosis and define the most appropriate tool to do so. A recent study comparing the GLIM malnutrition diagnosis with and without prior nutritional screening raised concerns about the risk for false negatives, since some of the patients (*n* = 11) were not identified as at nutritional risk by the RFH‐NPT, but were classified as malnourished according to the GLIM criteria.^26^

To validate the diagnosis of malnutrition by the GLIM criteria, the concurrent and/or predictive validity should be tested.^9^, ^10^ The SGA is widely used in patients with cirrhosis, and has served as the semi‐gold standard tool in the five studies in which the agreement or concurrent validity were tested and showed the lack of satisfactory validity for the GLIM criteria.^22^, ^23^, ^24^, ^29^, ^32^ This result may be partially justified by the different approaches used to assess the nutritional status by these two tools, since the SGA encompasses clinical reasoning, and the GLIM criteria are mostly based on objective parameters.^6^, ^7^ Regarding predictive validity, mortality was the most frequently assessed clinical outcome in studies with patients with cirrhosis.^18^, ^19^, ^20^, ^23^, ^29^, ^32^ The majority of studies demonstrated an independent association between malnutrition identified by the GLIM criteria and an increased risk of mortality,^18^, ^19^, ^20^, ^23^, ^29^ and other clinical outcomes.^19^, ^24^, ^25^ These results demonstrate that malnutrition according to the GLIM criteria is often associated with negative clinical outcomes in patients with cirrhosis.

In summary, from a clinical perspective, the GLIM framework can support the identification and diagnosis of malnutrition in patients with cirrhosis, particularly through the objective assessment of muscle mass. Most studies used anthropometric measures to estimate muscle mass, with mid‐arm muscle circumference showing a strong and consistent association with malnutrition and adverse outcomes while remaining inexpensive and feasible for routine clinical use. Moreover, with the availability of cutoffs and BMI‐based adjustment factors for mid‐upper arm circumference,^45^ this parameter can be further evaluated in this population across both research and clinical settings. In contrast, low BMI and weight loss are less reliable due to reduced accuracy in the presence of fluid overload. Health professionals should therefore select methods for assessing muscle mass carefully and interpret results in the context of fluid retention and disease severity. Furthermore, all phenotypic and etiologic criteria should be assessed to establish a diagnosis of malnutrition according to the GLIM framework.

This review has several limitations. The search was limited to studies published before January 2025 and written in English, which may have limited our findings. In addition, we did not assess the risk of bias of the included studies, and all the review processes were conducted by a single author, which may have increased the risk of bias. Also, although the GLIM criteria were recently proposed and should be adequately validated and even adjusted in the future, authors of some of the studies have considered this framework as the gold standard for evaluating other tools.^21^, ^24^, ^27^, ^28^, ^30^, ^34^ This approach is inadequate, since the main purpose of the framework is to standardize malnutrition diagnosis, and it should be complemented by other validated tools when necessary.^6^, ^7^ Due to the fact that the GLIM framework was often considered the gold standard, there are limited data about its concurrent validity in patients with cirrhosis so far, which precludes definite conclusions about this important aspect.

## CONCLUSIONS

Evidence about the use of the GLIM criteria for diagnosing malnutrition in patients with cirrhosis has demonstrated a lack of concurrent validity according to the GLIM working group standards, and adequate predictive validity. Authors of a few studies have assessed the concurrent validity as recommended, which makes it challenging to draw definite conclusions at the moment. The studies also differed substantially in terms of the methods applied to assess the muscle mass and disease burden/inflammation. Thus, authors should be aware of these gaps when designing future studies and should carefully review the publications from the GLIM working group on the guidance for assessing the individual criteria and the proper validation of the framework.^9^, ^10^ Furthermore, all five criteria should be assessed using accepted parameters, and the evidence available so far about the different combinations should help define the most appropriate tools. Finally, the sample size should be calculated to ensure that the sample has adequate power to demonstrate the effects being tested.

## AUTHOR CONTRIBUTIONS

**Lucilene Rezende Anastácio:** Conceptualization; methodology; supervision; writing—review and editing. **Bárbara Chaves Santos:** Conceptualization; methodology; investigation; writing—original draft. **Ana Teresa Limon‐Miro:** Writing—review and editing. **Puneeta Tandon:** Writing—review and editing. **Maria Isabel Toulson Davisson Correia:** Writing—review and editing. **Carla M. Prado:** Writing—review and editing. **Maria Cristina Gonzalez:** Writing—review and editing.

## CONFLICT OF INTEREST STATEMENT

Maria Cristina Gonzalez has previously received honoraria and/or paid consultancy from Abbott Nutrition, Nutricia, and Nestlé Health Science Brazil. Carla M. Prado has received honoraria and/or paid consultancy from Abbott Nutrition, Nutricia, Nestlé Health Science, and Novo Nordisk. All other authors declare no conflicts of interest.
